# A prospective, single-arm, phase II clinical trial of intraoperative radiotherapy using a low-energy X-ray source for local advanced Laryngocarcinoma (ILAL): a study protocol

**DOI:** 10.1186/s12885-020-07233-1

**Published:** 2020-08-06

**Authors:** Yining Yang, Li Li, Yongzhe Zheng, Qingfeng Liu, Xianfeng Wei, Xinyuan Gong, Wei Wang, Peng Lin

**Affiliations:** 1 Department of Radiotherapy and Department of Otorhinolaryngology Head and Neck Surgery, Tianjin First Central Hospital, No.24 FuKang Road, Nankai District, Tianjin, 300192 China; 2 Institute of Otolaryngology of Tianjin, Tianjin, China; 3 Key Laboratory of Auditory Speech and Balance Medicine, Tianjin, China; 4 Key Clinical Discipline of Tianjin (Otolaryngology), Tianjin, China; 5 Otolaryngology Clinical Quality Control Centre, Tianjin, China; 6 Department of Radiotherapy, Tumor Hospital of the Chinese Academy of Medical Sciences, Beijing, China

**Keywords:** Local advanced laryngocarcinoma, IORT, Low-energy X-ray, Local control

## Abstract

**Background:**

Laryngocarcinoma (LC), in most cases a squamous cell carcinoma, accounts for 1 ~ 5% of the incidence of all tumors. At present, laryngocarcinoma is mainly managed with the integration of surgery and radio- and chemo-therapies. The current development trend of treatment is to improve the local control rate of tumor and the quality of life of patients. Intraoperative radiation therapy (IORT) is a radiotherapy that delivers single high dose irradiation at a close range to the tumor bed during the surgical operation process. It has particular radiobiological advantages in protecting normal surrounding tissues by directly applying the irradiation dose to the high-risk tumor bed area.

Two forms of IORT, i.e., high dose rate (HDR) brachytherapy and external beam radiotherapy (EBRT, including electron and photono IORT), had been studied before the treatment of head and neck tumors (including laryngocarcinoma). However, no relevant assessment had been carried out on 50KV low-energy X-ray. We are convinced by certain arguments that the application of low-energy X-ray for intraoperative local radiotherapy of laryngocarcinoma can not only achieve the therapeutic effect of IORT but also reduce the incidence of high-energy irradiation related toxic and side effects. The purpose of this study is to observe the safety and short-term efficacy of IORT when used in conjunction with standard of care for the treatment of local advanced laryngocarcinoma (LAL).

**Methods/design:**

In consideration of the applications of precise targeted IORT in oncosurgery and in line with the application range and reference clinical medical guidances approved by SFDA (ZEISS radiosurgical operation system has been used for the treatment of solid tumors since 31 December, 2013 with an approval from SFDA), we have preliminarily planned the tumors suitable for IORT, determined the members of MDT in our hospital, improved the MDT diagnosis and treatment processes for the tumors, established the standards, indications and contraindications for the application of IORT, determined the indicators to be observed after the treatment of tumors with surgical operations plus IORT, and carried out follow-up visits and statistical analysis.

This is a single-arm, prospective Phase II clinical trial of the treatment of LAL patients with IORT + EBRT. The study subjects are followed up for statistics and information of their acute/chronic toxic reactions and local control rate, DFS, and OS etc. The safety and short-term efficacy of the application of IORT as SIB for the treatment of LAL. The sample size of the study is 125 subjects.

**Discussion:**

The safety and efficacy of IORT for the treatment of head and neck cancers have been proven in studies by multiple institutions (1–3). The purpose of this study is to investigate the maximum safe dose and short-term efficacy of IORT for providing a theoretical basis for clinical trials.

**Trial registration:**

Trial registration: Clinicaltrials.gov, NCT04278638. Registered 18 February 2020 - prospectively registered, https://clinicaltrials.gov/ct2/show/NCT04278638

## Background

The incidence of laryngocarcinoma accounts for 1–5% of the incidence of all tumors. According to Global Cancer Statistics 2018, there were nearly 200,000 new laryngocarcinoma patients and approximately 100,000 cases of laryngocarcinoma-related deaths in 2018 worldwide [1]. According to NCCN Guidelines for Head and Neck (H&N) Cancers (2019.V1) [2], postoperative radiotherapy/chemotherapy may be necessary and recommended depending on the condition of the tumor after surgical treatment of LAL. A meta analysis of the treatment of LAL with surgical operation alone or the combination of surgery and radiotherapy/chemotherapy yielded results suggesting that, the combination of surgery and postoperative adjunctive radiotherapy probably had better efficacy and safety than those of surgery alone or the combination of surgery and postoperative chemotherapy [3]. However, the study also suggested that the timing of postoperative radiotherapy was of important significance to the treatment of tumors and the delay in postoperative EBRT might have adverse impact on the treatment result of tumors [4, 5].

IORT is a technique for intraoperative delivery of single high dose irradiation at a close range to the tumor bed, residual lesions, lymphatic drainage areas and potential invaded areas after tumor resection [6]. The simultaneousness of IORT and surgery enables intraoperative precise setting of irradiation field and effectively reduces the time interval between surgery and radiotherapy. With IORT, radioactive rays can be delivered to the surface of tumor bed for effectively limiting the delivery of large dose radiation to normal surrounding tissues [7] and achieving higher biological effect than routine SIB (1.26 ~ 1.42) [8]. In light of this, IORT has indubitable advantages.

Nowadays IORT has been extensively used for the treatment of breast cancer, pancreatic cancer, head and neck tumors, thoracic and abdominal tumors and tumors at other body sites [9, 10]. The safety and efficacy of IORT for the treatment of head and neck tumors have been proven in studies by multiple institutions, therefore, IORT can be used for SIB to optimize the local control of tumors [11]. IORT has become one of the most commonly used and effective therapy regimens, especially in the treatment of patients with recurrent head and neck tumors [12]; it also has apparent advantages in the treatment of patients at high risk of recurrence, patients with serious or minor residual disease(s), patients underwent improvement and/or salvage surgery, and patients with history of EBRT. It has been reported in literature that the probability of complication(s) arising from IORT of head and neck tumors is clinically acceptable. Toita et al. had demonstrated in their early-stage studies that the incidence of toxic reactions would significantly increase when the irradiation dose exceeded 20Gy [13]. There were other studies suggesting that the incidence of toxic reactions varied significantly with different dose [14, 15]. Of the complications that had been reported, carotid artery rupture had an incidence between 2 and 5% [11, 16, 17]. Carotid artery rupture is a treatment complication associated with the highest mortality. The incidence of fistula/abscess is in the range of 4% ~ 15% [11, 14, 15, 17–19]. The incidence of osteoradionecrosis is in the range of 0 ~ 13% [11, 13–15, 20, 21]. Furthermore, some studies reported that the incidence rate of treatment-related neuropathy was in the range of 1% ~ 3% [18, 21, 22]. It should be noted that an MSKCC report pointed out a study on the prognosis of 57 patients with recurrent tumors revealed that the incidence of neuropathy was 26% and the incidence of fibrosis was 29% [21]. It is noteworthy that the above-mentioned studies used different toxicity scales and their median follow-up visit period varied. For experienced centers, IORT is an effective therapy that has reasonable toxicity, does not increase perioperative mortality rate or hospitalization duration, and yields satisfactory outcomes with local controlled treatment [15, 18, 22]. During the treatment of patients with local advanced head and neck tumors, the delay in postoperative EBRT may have adverse impact on the treatment result of tumors [18, 23] In contrast, IORT for its ability to deliver instantaneous tumor bed targeted irradiation to patients during their surgery process and better selectivity for target tissues can reduce the irradiation dose delivered to the patients. One of the potential benefits of IORT is that it can minimize the time interval between surgery and radiotherapy during the treatment of LAL, as there were studies showing that the delay of radiotherapy would affect the prognosis of LC [19, 24]. IORT plays an important role in the treatment of local advanced head and neck tumors since it administers SIB to microscopic residual tumor or residual tumor tissues in the vicinity of important structures whereas a negative margin is not a guarantee of no recurrence.

In spite of the apparent advantages of IORT in the treatment of head and neck tumors, the application of IORT for the treatment of local advanced head & neck tumors is still in exploratory stage. In particular, there is a shortage of studies evaluating the efficacy of IORT in the treatment of LAL. Judged from the results reported in literature, the probability of complications after IORT of local advanced head and neck tumors is clinically acceptable. This study aims to investigate the safety and short-term treatment results of IORT as postoperative SIB radiotherapy of LAL and further detail and verify the assessment of toxicity and efficacy of IORT for the treatment of LAL. This study verifies the following hypothesis: compared with the current standard of care, IORT can improve local control, as observed in the patients in the current study.

## Methods

**Clinical trial stage:** Phase II.

**Design**: single-center, single-arm, prospective clinical study.

### Purpose

This is a single-center, single-arm, prospective clinical trial intended to investigate the safety and short-term therapeutic effect of IORT as a postoperative simultaneous integrated boost (SIB) radiotherapy for the treatment of LAL. The trial has been registered at www.clinicaltrials.gov (NCT04278638).

### Main objectives

1. Feasibility and safety of IORT irradiation dose for the treatment of LAL;

2. 2-year local recurrence rate;

3. Acute and chronic toxicity, 2-year DFS, PFS and OS.

### Outcome indicators

Primary endpoint: local recurrence rate 2 years after the radiotherapy.

Secondary endpoints: DFS and OS after the radiotherapy.

Safety indicators: acute and chronic toxicological reactions (necrosis and fibrosis of local tissues) after IORT SIB, wound healing time, wound infection, wound rupture, pharyngeal fistula, radiation-induced pain, etc.

### Study subjects

Subjects of this study are patients with histologically confirmed LAL who have received total- or hemi-laryngectomy and postoperative radiotherapy and signed informed consent form at Tianjin First Central Hospital. Subjects in the treatment group are enrolled from 1 January 2019 to 1 July 2020.

### Inclusion criteria

1. Adults aged over 18;
2. Pathologically diagnosed with LAL (T2N1–3 / T3N0–3 / T4N0–3);
3. With surgical indications of total- or hemi-laryngectomy;
  - Without remote metastasis
  - Resectable tumor margin
  - Without involvement of any important organ/tissue (esophagus, blood vessels)
4. Survival time expectation ≥3 months;
5. With insight for signing informed consent forms for treatment and study.

### Exclusion criteria

1. With concomitant involvement of important surrounding tissues (blood vessels, esophagus), which may give rise to serious complications such as life-threatening angiorhagia, post-chemotherapy esophageal ulcer or stenosis that is hard to heal;
2. With remote metastasis;
3. With multiple primary cancers;
4. With surgical tumor bed that is unsuitable for IORT;
5. Pregnant or planned to pregnant;
6. With contraindication(s) of follow-up visit CT/MRI examinations;
7. Unable to receive treatment and/or follow-up visits as scheduled;
8. With cachexia, organ function decompensation;
9. Participating in other clinical trial.

### Examination method

Examinations of the study subjects include physical examination, laboratory tests (blood routine examination, biochemistry), laryngendoscopy, imaging examinations (CT or MRI examination of larynx) and, if necessary, histological examination and positron emission tomography (PET).

### Sample size

The primary purpose of this study is to investigate the safety and efficacy of IORT as SIB therapy that directly acts on surgical tumor bed in place of conventional EBRT for the treatment of LAL; the primary study endpoint is the local recurrence rate 2 years after the treatment of LAL with surgery in combination with IORT. This is a single-center, single-arm clinical study. In our center, the 2-year local recurrence rate of LAL treated with conventional therapeutic regimen is approximately 20%; the 2-year local recurrence rate of laryngocarcinoma treated with surgery in combination with IORT reported in literature is approximately 15–70% [14, 25]. In light of the above factors, the local recurrence rate of laryngocarcinoma treated with IORT as SIB therapy in this study is assumed to be 10% (10% lower than conventional therapeutic regimen’s). The hypothesis is tested using a 2-sided test at the alpha = 0.05 level of significance with a power of 80%. The required sample size of the study is 107 subjects. Based on a 15% lost to follow-up rate, the total sample size of this study is 125 subjects.

### Study procedures

1. Screening patients with LAL;

2. Signing informed consent forms for surgery and IORT;

3. Hemilaryngectomy/total laryngectomy under general anesthesia + IORT;

4. Carrying out follow-up visits every day during the hospitalization after the surgery and every 1–3 months after discharge (Table 1).

**Table 1:** Follow-up visit schedule (month)

	1	3	6	9	12	18	24	30	36
Acute toxicological reactions	√	√							
Chronic toxicological reactions			√	√	√	√	√	√	√
Efficacy indicators		√	√	√	√	√	√	√	√

Therapeutic regimens:

1) Resectable range of tumor.

a) Total laryngectomy; b) Tumor bed IORT; c) EBRT.

2) Range of tumor resectable and laryngeal function retainable.

a) Hemilaryngectomy; b) Tumor bed IORT; c) EBRT.

### IORT technology

Name: IORT radiosurgical treatment system.

Trade Name: INTRABEAM®PRS500 System.

Manufacturer: Carl AG, Germany.

### IORT treatment pathway

**Fig. 1:**
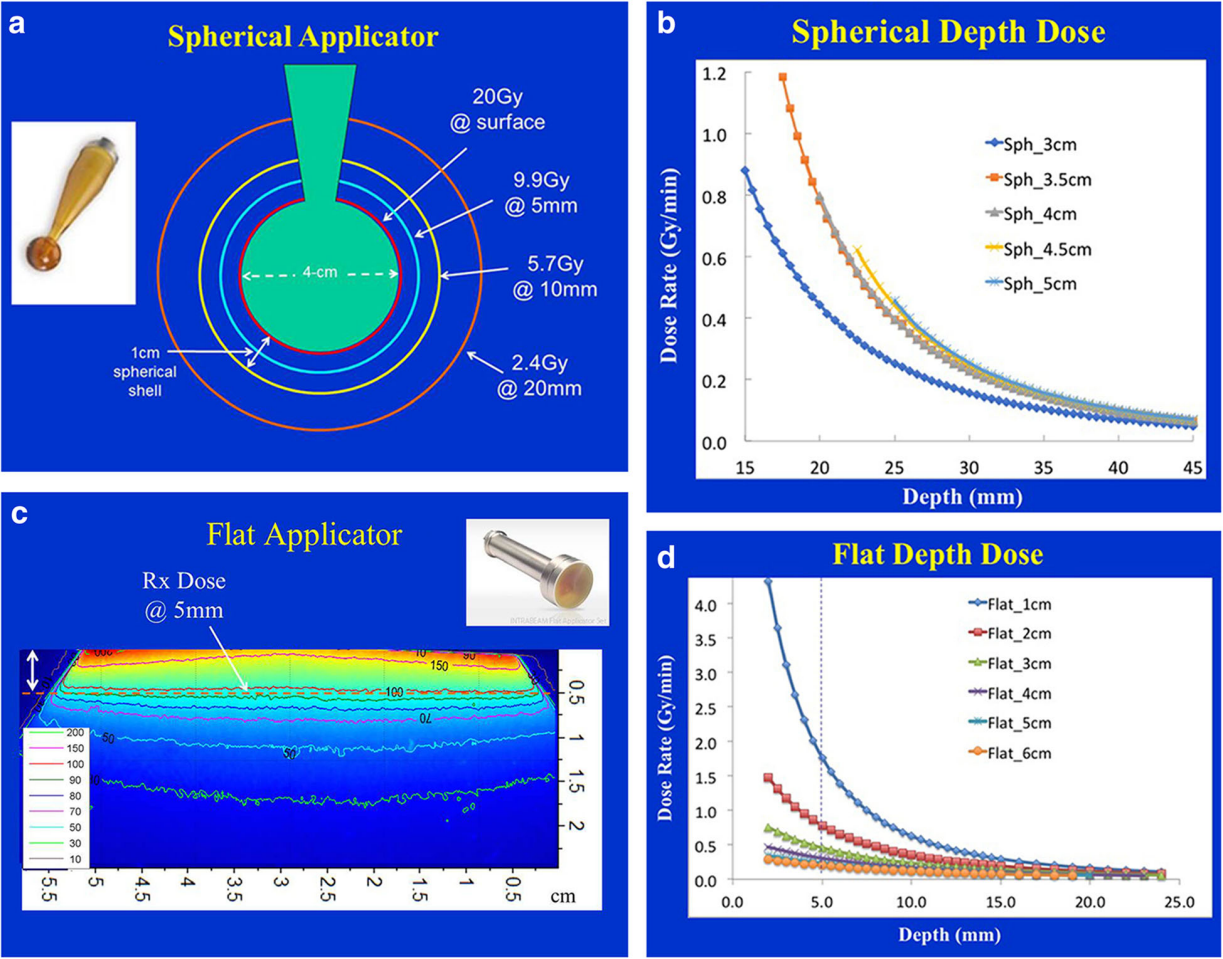
Intraoperative interstitial irradiation dosiology

1. Laryngocarcinoma under general anesthesia
2. A spherical applicator of properly selected diameter is placed by the surgeon under direct vision and measurement to the postoperative tumor bed area
  **Hemi laryngectomy + use of spherical applicator at tumor bed.**
  **Total laryngectomy + use of plate applicator at tumor bed**
3. An appropriate irradiation dose is selected by the radiotherapist in order to protect normal surrounding tissues (Fig. 1) [26].

### Duration of surgical treatment

Surgical operation (surgery under anesthesia) ≤3 h.

### Operation procedure

**Fig. 2:**
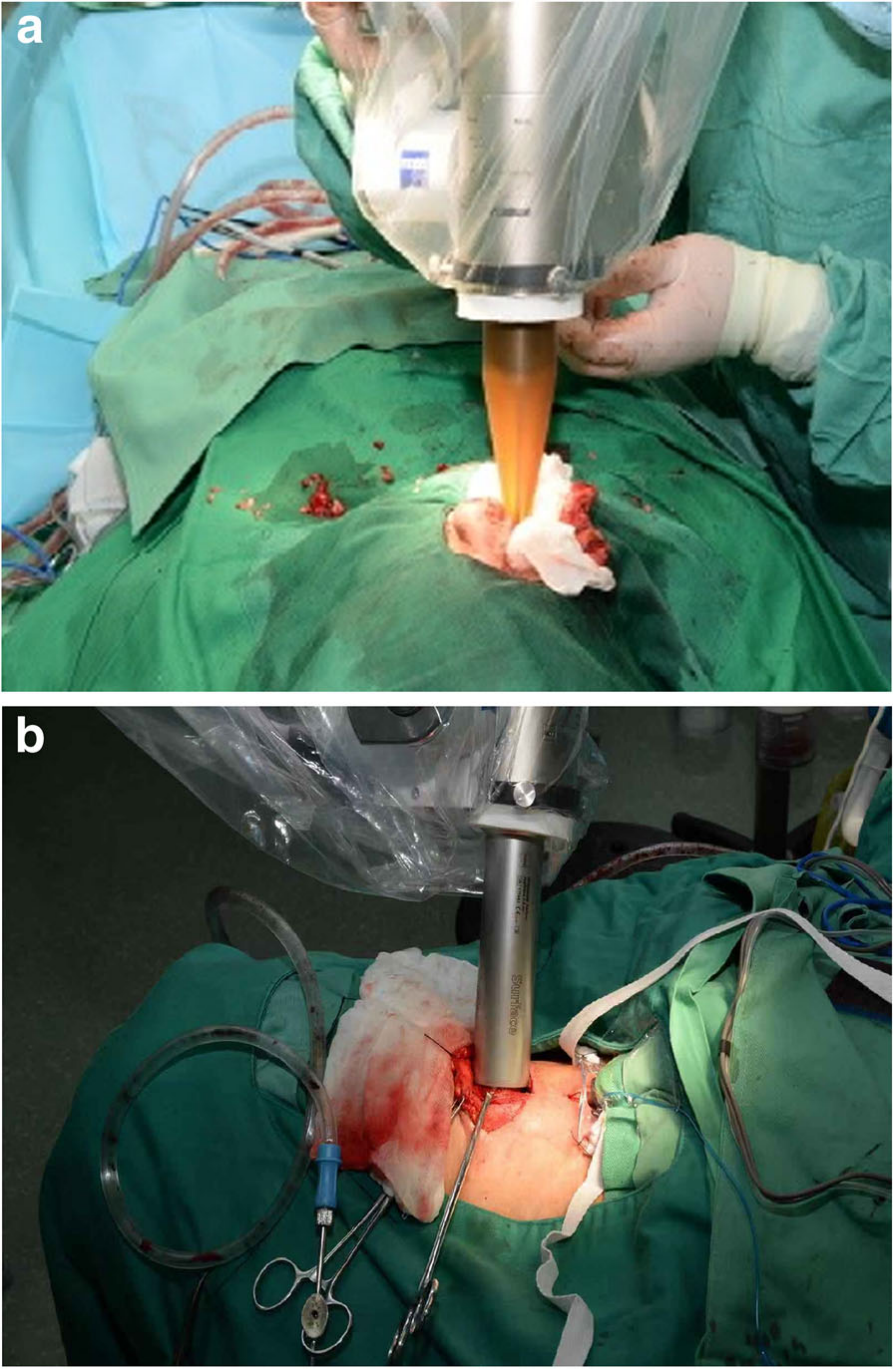
The Intrabeam IORT procedure

Connect a mobile 50 KV X-ray radiation source (Intrabeam) to the mechanical arm and maintain the source’s stability throughout the treatment process (Fig. 2). Before each treatment, calibrate and verify the IORT system, including the calibration of probe alignment of the IORT control system, dynamic deviation of electron beam, isotropism and output dose. IORT target volume shall include any area on tumor bed deemed at risk by the surgeon and radiologist, an applicator of appropriate diameter (3.0, 3.5 or 4.0 cm) and probe shall be selected in accordance with the target volume, and the application shall be fixed to the X-ray source probe. Cover the sterile drape over the IORT equipment to prevent contamination. The applicator shall be jointly confirmed by the radiologist and the surgeon in order to ensure the normal surround tissues and tissues sensitive to radiation are not in the treatment area. When the equipment is locked at treatment site, a single irradiation shall be delivered at a dose of 10 Gy (or 12Gy) to the corresponding target volume at specified depth.

### Follow-up visits of subjects

The study subjects shall be followed up for 2 years after the treatment with surgery in combination with IORT for examination and assessment of the study subjects’ surgical wound and the first follow-up visit shall be done 1 month after the treatment. The frequency of regular follow-up visits shall be changed to once every 3 months from the third month onwards and once every 6 months from the second year onwards. The follow-up of the study subjects shall include physical examination, wound examination, and regular CT or MRI examination.

Local recurrence: CT or MRI reexamination shows typical radiographic features of local recurrence.

DFS: From subject’s enrollment for treatment until his/her first local recurrence or remote metastasis or other non-study-related death.

OS: From subject’s enrollment for treatment until his/her death.

### Main result indicators: safety

- Assessment of IORT-related toxic and side effects

✓ Wound healing time

✓ Infection

✓ Pharyngeal fistula

✓ RT-related mucosal reactions

✓ Fibrosis of mucosa

✓ RT-related toxic and side reactions of laryngeal nerve

### Statistical analysis

This study implemented strict quality control in every aspect of the baseline and follow-up surveys, including the development of a unified questionnaire, the study protocol operation manual, etc.; all personnel in the baseline and follow-up surveys underwent strict training and technical examination before participating in the study; the field survey form was checked and verified by quality control personnel, and all data were double-entry.

The primary endpoint of this study is local recurrence rate. The comparison of the local recurrence rate between subjects of this study and subjects treated with conventional therapeutic regimen shall be done with Chi-square test. The comparison of time to local recurrence shall be done with Kaplan-Meier survival curve and log-rank test. During the comparison of local recurrence rate, confounding variables shall be controlled with proportional hazards model.

### Technical route

Figure 3

**Fig. 3:**
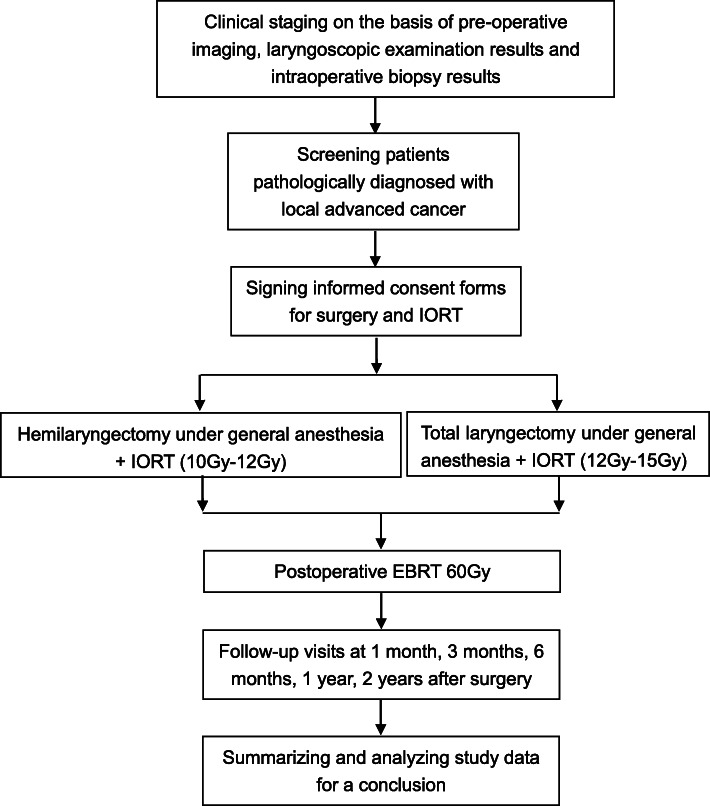
Treatment schedule

## Discussion

Head and neck tumors are generally treated with an integrated treatment modality by a multi-disciplinary team (MDT). The precise application of a variety of therapies is an important component of individualized treatment. Postoperative prophylactic irradiation after LAL surgery can reduce the disease’s recurrence rate from 20 to 50% to 18–19%. This study aims to investigate whether the addition of low-energy X-ray source IORT to the standard of care of LAL for instantaneous SIB featuring high precision, high dose, and high biological effect can be effectively used in place of EBRT in order to reduce the local recurrence rate of LAL.

The treatment idea of IORT is in line with the concept evolution from radical surgery to risk assessment based minimally invasive modified surgery in oncosurgery. Compared with conventional EBRT, IORT can precisely set the irradiation field for direct destruction of unresectable and/or residual tumor tissues with mild low-energy X-ray (50 kV) irradiation of tumor bed at shallow irradiation depth. The technique can increase the effective irradiation dose on local tumor bed without causing significant damage to normal tissues by virtue of its ability to evade the irradiation of dose-limiting sensitive tissues partially or completely. In other words, a single intraoperative large dose irradiation can destroy the micro-environment of tumor more effectively than conventional radiotherapy.

IORT and conventional EBRT can mutually make up for each other’s shortcomings: the shortcoming of conventional radiotherapy, i.e., its inability to deliver high irradiation dose to target area due to the restriction of sensitive surrounding tissues/organs, can be addressed by IORT; the shortcomings of IORT, i.e., the non-uniform irradiation and limited irradiation unit dose during its application in target areas of irregular shapes, can be remedied by EBRT. Therefore, the combination of IORT and postoperative conventional irradiation can achieve ideal results featuring high irradiation dose in tumor target areas and complete irradiation dose coverage in areas at risk.

Considering the huge potential of IORT for improving local control, we are looking forward to use this study to evaluate the effect of IORT in the treatment of LAL.

## Data Availability

The raw/processed data required to reproduce these findings cannot be shared at this time as the data also forms part of an ongoing study.

## Abbreviations

EBRT: External beam radiotherapy
HDR: High dose rate
IORT: Intraoperative radiation therapy
LAL: Local advanced laryngocarcinoma
LC: Laryngocarcinoma
MDT: Multi-disciplinary team
PET: Positron emission tomography
SIB: Simultaneous integrated boost
